# Mitochondrial function is impaired in the skeletal muscle of pre-frail elderly

**DOI:** 10.1038/s41598-018-26944-x

**Published:** 2018-06-04

**Authors:** Pénélope A. Andreux, Marcus P. J. van Diemen, Maxime R. Heezen, Johan Auwerx, Chris Rinsch, Geert Jan Groeneveld, Anurag Singh

**Affiliations:** 1 Amazentis SA, EPFL Innovation Park, Batiment C, CH-1015 Lausanne, Switzerland; 2 Center for Human Drug Research, Zernikedreef 8, 2333 CL Leiden, The Netherlands; 3 Laboratory for Integrative and Systems Physiology, Ecole Polytechnique Fédérale de Lausanne, CH-1015 Lausanne, Switzerland

**Keywords:** Mitochondria, Predictive markers

## Abstract

Aging is accompanied by a gradual decline in both muscle mass and strength over time, which can eventually lead to pathologies, such as frailty and sarcopenia. While these two conditions are well characterized, further investigation of the early biological signs present in pre-frail elderly is still needed to help identify strategies for preventative therapeutic intervention. The goal of the present clinical study was to evaluate the level of mitochondrial (dys)function in a well-defined population of pre-frail elderly (>60 years of age). Pre-frail elderly were compared with an age-matched population of active elderly. Muscle mitochondrial function was assessed *in vivo* using phosphorus magnetic resonance spectroscopy (^31^P-MRS) and a comprehensive set of biological biomarkers were measured *ex vivo* in vastus lateralis muscle biopsies. In pre-frail subjects, phosphocreatine recovery was impaired and mitochondrial respiratory complex protein and activity levels were significantly lower when compared with active elderly. Analysis of microarray data showed that mitochondrial genes were also significantly down-regulated in muscle of pre-frail compared to active elderly. These results show that mitochondrial impairment is a hallmark of pre-frailty development and the onset of decline in muscle function in the elderly.

## Introduction

Population based studies have demonstrated that approximately half of the elderly population displays early signs of either muscle mass or function decline^1^. If left unaddressed, the muscle health of elderly can deteriorate further, manifesting as frailty and sarcopenia, two geriatric conditions that have a tremendous health economic impact^2^. Today, one way of diagnosing frailty in the clinic is to use Fried’s criteria, which consists of five components: unintentional weight loss, general fatigue, decline in muscle strength, slow gait speed and low physical activity^3^. Elderly exhibiting three or more of these criteria are classified as frail, while those experiencing one or two Fried’s criteria are classified as pre-frail^3^. Sarcopenia is diagnosed based on the gradual loss of muscle mass leading to a decline in muscle strength, with muscle atrophy being the hallmark feature in elderly with sarcopenia^4^. As life expectancy increases in society with 1 out of every 5 people in the world expected to be elderly (>60 years) by 2050^5^, maintaining muscle health will be key to ensure an independent and unassisted lifestyle in the elderly population. Further understanding of the biological causes driving age-related muscle decline is therefore needed.

From a broader perspective, most aging cells progressively lose their capacity to maintain their optimal function, ultimately leading to organ specific symptoms and the development of systemic age-related diseases. This time-dependent phenomenon is currently thought to result from the accumulation of damage both at the DNA and protein levels^6^. One of the most sensitive organelles to these changes is the mitochondrion. Research in a wide range of preclinical models has shown that mitochondrial bioenergetics, defined as the capacity of the mitochondria to respond to the energetic and metabolic demand of the cell, is declining with age^7,8^. Furthermore with aging there is a reduction in mitochondrial turnover due to lower biogenesis and damaged mitochondria being less effectively cleared by mitophagy^6,9^. Previous clinical studies have reported associations with low mitochondrial abundance and impairment in mitochondrial function in sedentary elderly when compared to either young or age matched active elderly subjects^10,11^. Some evidence also points to decreased mitochondrial function in frail elderly immobilized due to hip fracture and in clinically diagnosed sarcopenia^12,13^. However, to our knowledge, the level of mitochondrial (dys)function in pre-frail elderly (i.e. the beginning phase of the symptomatic decline in muscle and physical performance associated with aging) has previously not been well characterized.

In the current clinical study, age-matched pre-frail elderly were compared to active elderly with respect to the status of their muscle mitochondrial function *in vivo* using phosphorus magnetic resonance spectroscopy (^31^P-MRS) and a comprehensive set of biological biomarkers, measured *ex vivo* in *vastus lateralis* muscle biopsies. The results of this investigation demonstrate a striking association of pre-frailty status in elderly with mitochondrial impairment in skeletal muscle and provide a strong rationale for employing interventions that improve mitochondrial function to either reverse the pre-frailty phenotype or delay the progression to full onset frailty syndrome.

## Results

### Demographics

**Figure 1:**
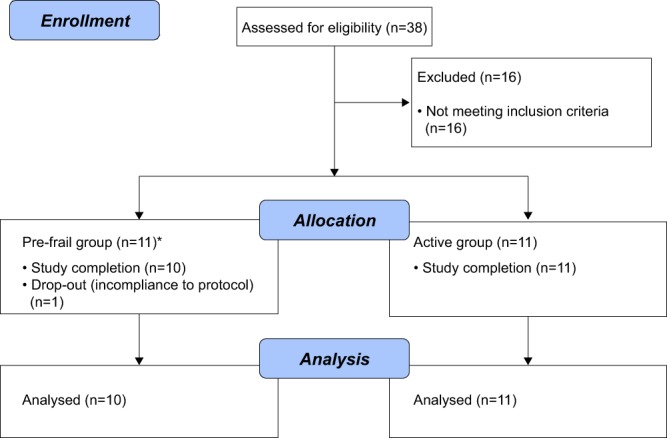
Flow diagram of the subject enrollment and analysis. Subjects were screened for eligibility up to 45 days before study enrollment. Out of the 38 elderly subjects assessed, 22 were included into the study of which 11 were pre-frail and 11 were active elderly. 21 subjects completed the study and were included in the final analysis. One pre-frail subject who was non-compliant with the study protocol dietary restriction between the visits on Day 1 and Day 14 was excluded from all analysis except the genomic expression via microarray (^∗^as muscle biopsy had already been collected on Day 1), and was considered a drop-out.

In total, 11 pre-frail (6 males and 5 females) and 11 active (6 males and 5 females) subjects between the ages of 61 to 80 years old participated in this study. One pre-frail male subject was excluded from the final study analyses, due to a lack of compliance to study dietary restrictions between visits on Day 1 and Day 14 (Fig. 1). However, because he had already been collected for muscle biopsy on Day 1, he was included for the microarray genomic analysis only. In the end, data from 10 pre-frail (5 males and 5 females) and 11 active (6 males and 5 females) subjects were included for all analysis except gene expression via microarray. Pre-frail and active subjects were matched on age (70.2 ± 5.8 *vs* 70.0 ± 6.7 yrs) and body mass index (BMI) (25.7 ± 4.2 *vs* 24.6 ± 3.9 kg/m^2^) (Table 1). Subjects were all of Caucasian descent, except for one active subject, who was of Afro-Dutch descent (Table 1).

**Table 1:** Subject demographics.

Demographics	Pre-frail (n = 10)	Active (n = 11)
Age, mean (SD), range, in years	70.2 (5.8), 61–80	70.0 (6.7), 63–78
Sex, n (%)		
Male	5 (50)	6 (55)
Female	5 (50)	5 (45)
Race, n (%)		
Caucasian	10 (100)	10 (91)
Afro Dutch	0 (0)	1 (9)
Body Mass Index, mean (SD), range, in kg/m^2^	25.7 (4.2), 17.8–33.2	24.6 (3.9), 20.1–32.0

### Muscle function and physical performance measures

As described in the methods section (inclusion/exclusion criteria), subjects were selected as pre-frail when they fulfilled at least one to two out of the three following Fried criteria^4^, i.e. muscle weakness (low muscle strength), slow gait speed and low physical activity. All subjects analyzed in the pre-frail population in the end met at least two of the Fried frailty criteria. Pre-frail subjects had low physical activity with a mean energy expenditure of 392 MET per week, which corresponds to less than 20 minutes of walking per day. In comparison, the mean energy expenditure in the active elderly group was 6,508 MET per week (p = < 0.0001 when compared to pre-frail group), which corresponds to 1 hour of vigorous exercise per day. There was a slight trend, though not statistically different, in the decline of skeletal muscle mass index in elderly (10.9 kg/m^2^ in pre-frail *vs* 12.1 kg/m^2^ in active elderly, p = 0.21), highlighting, at least in these populations, that differences in muscle performance were not strictly linked to muscle mass, but were driven through other changes in the muscle biology.

Upon inclusion in the study, four additional tests were performed at day 1 and day 14: 1) SPPB, 2) handgrip strength by Jamar dynamometry, 3) quadriceps strength, 4) postural stability and 5) *in vivo* evaluation of mitochondrial function using phosphorus magnetic resonance spectroscopy (^31^P-MRS) (Table 2). All these tests were very reproducible from one visit to another (data not shown). Therefore, the average value between the two visits per subject was used to compute statistical significance. No differences were observed in the total SPPB score when comparing the two groups (total mean SPPB score of 10 in pre-frail subjects *vs* 11 in active elderly, p = 0.58; Table 3). There was no difference in the balance test performed during the SPPB (total score of 3 compared to 3.18 in the active elderly, p = 0.33, Table 3), as well as the postural stability test (383 compared to 392 in the active elderly, p = 0.88, Table 3). However, when looking at the other individual SPPB domain tests, there were significant differences in the pre-frail group for the duration sit-to-stand transfer test: 13.24 seconds compared to 9.77 seconds in the active group (p = 0.03) and for the gait-speed: 0.99 m/s as compared to 1.40 m/s in the active group (p = 0.0088). Pre-frail elderly clearly exhibited low muscle strength, with a handgrip strength of 22.3 kg *vs* 38.8 kg in the active group (p = 0.0002) and a quadriceps strength of 141 Newton *vs* 223 Newton in the active group (p = 0.0002) (Table 3). Altogether, these results show that the screening parameters were consistent and led to a clear selection of two distinct populations.

**Table 2:** Study schedule.

Test	Screening	Visit 1 (Day 1)	Visit 2 (Day 14)
International Physical Activity Questionnaire (IPAQ)	×		
Body composition by bioelectrical impedance analysis	×		
Short Physical Performance Battery (SPPB) test	×	×	×
Handgrip strength by Jamar dynamometry	×	×	×
Quadriceps strength		×	×
Postural stability		×	×
^31^P-Magnetic Resonance Spectroscopy (^31^P-MRS)		×	×
Muscle biopsy (*vastus lateralis*)		×

**Table 3:** Subject physical performance characteristics.

	Pre-frail (n = 10)	Active (n = 11)
**Parameters measured at screening**		
Physical activity in MET per week	392^∗∗∗^ (109.2), 262.0–579.0	6508 (5258.5), 2555–19344
Skeletal Muscle Index in kg/m^2^	10.90 (2.70), 4.94–13.61	12.10 (2.50), 8.82–16.00
**Parameters measured during the study (average between day 1 and day 14)**		
Handgrip strength in kg	22.3^∗∗∗^ (5.7), 12.4–31.2	38.8 (10.3), 23.9–52.3
Quadriceps strength in Newton meters	141^∗^ (55.97), 61–220	223 (51.93), 133–302
Short Physical Performance Battery		
*Total score*	10 (1.70), 7−12	11 (1.81), 8–12
*Gait speed in meters per second*	0.99^∗∗^ (0.22), 0.46–1.19	1.40 (0.20), 1.13–1.76
*Duration sit to stand transfer in seconds*	13.24^∗^ (6.32), 7.66–24.0	9.77 (2.70), 5.75–15.78
*Balance test*	3 (0), 3–3	3.18 (0.60), 3–4
Postural stability in mm sway	383 (386.85), 202.5–488.0	392 (413.37), 196.3–970.1

All values are given as mean (SD), range. *P < 0.05; **P < 0.01; ***P < 0.001 when comparing group means between pre-frail and active groups.

### Non-invasive measurement of mitochondrial function in muscle

**Figure 2:**
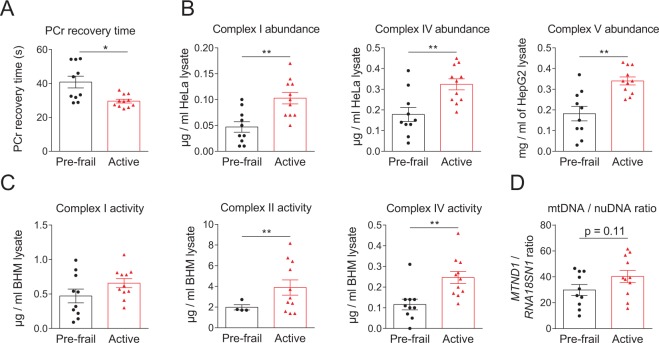
Mitochondrial function is lower in *vastus lateralis* of pre-frail subjects. (**A**) Phosphorus Magnetic Resonance Spectroscopy (^31^P-MRS) was used to measure PCr recovery time, a marker of mitochondrial function, in the right posterior calf muscle. (**B**) Mitochondrial respiratory complexes I, IV and V abundance in *vastus lateralis*. (**C**) Mitochondrial respiratory complexes I, II and IV activity in *vastus lateralis*. (**D**) Relative abundance of mitochondrial DNA (mtDNA) over nuclear DNA (nuDNA) measured by qPCR. The ratio was calculated by comparing the relative abundance of mitochondrial encoded NADH dehydrogenase 1 (*MTND1*) to the nuclear encoded gene RNA 18S ribosomal N1 (*RNA18SN1*). Data represent mean ± SEM. ^*^P < 0.05; ^∗∗^*P* < 0.01; ^∗∗∗^*P* < 0.001 after a two-tailed Student t test.

Mitochondrial function was first assessed in calf muscle using ^31^P-MRS by measuring phosphocreatine (PCr) recovery rate (Table 2). This parameter corresponds to the rate at which creatine is re-phosphorylated after exercise and is a function of the rate at which mitochondria produce ATP^14^. PCr recovery rate over both visits was found to be significantly longer in pre-frail subjects than in active subjects (40.82 seconds in pre-frail elderly *vs* 29.53 in active elderly; p = 0.0064, Fig. 2A), indicating a lower rate of ATP production in the muscle in pre-frail subjects. These results with ^31^P-MRS were reproducible during the two visits on Day 1 and 14 of the study.

### Measurement of mitochondrial respiratory complexes activity in muscle biopsies

Mitochondrial function was evaluated in muscle biopsies collected at day 1 of the study (Table 2), by measuring complexes I, IV and V abundance and complexes I, II and IV activity. Active subjects presented both higher abundance of complexes I, IV and V and higher enzymatic activity of complex I, II and IV than measured in pre-frail subjects (Fig. 2B,C). These results agree with the PCr recovery rate data and indicate that mitochondria in the muscle of active subjects are both more abundant and/or more efficient than those found in pre-frail subjects. Mitochondrial abundance was estimated by measuring the mitochondrial DNA (mtDNA) over nuclear DNA (nuDNA) ratio. There was a trend to have higher mtDNA/nuDNA ratio in active subjects compared to pre-frail subjects, which however was not significant (p = 0.11) (Fig. 2D).

### Transcriptomics analysis of skeletal muscle biopsies

**Figure 3:**
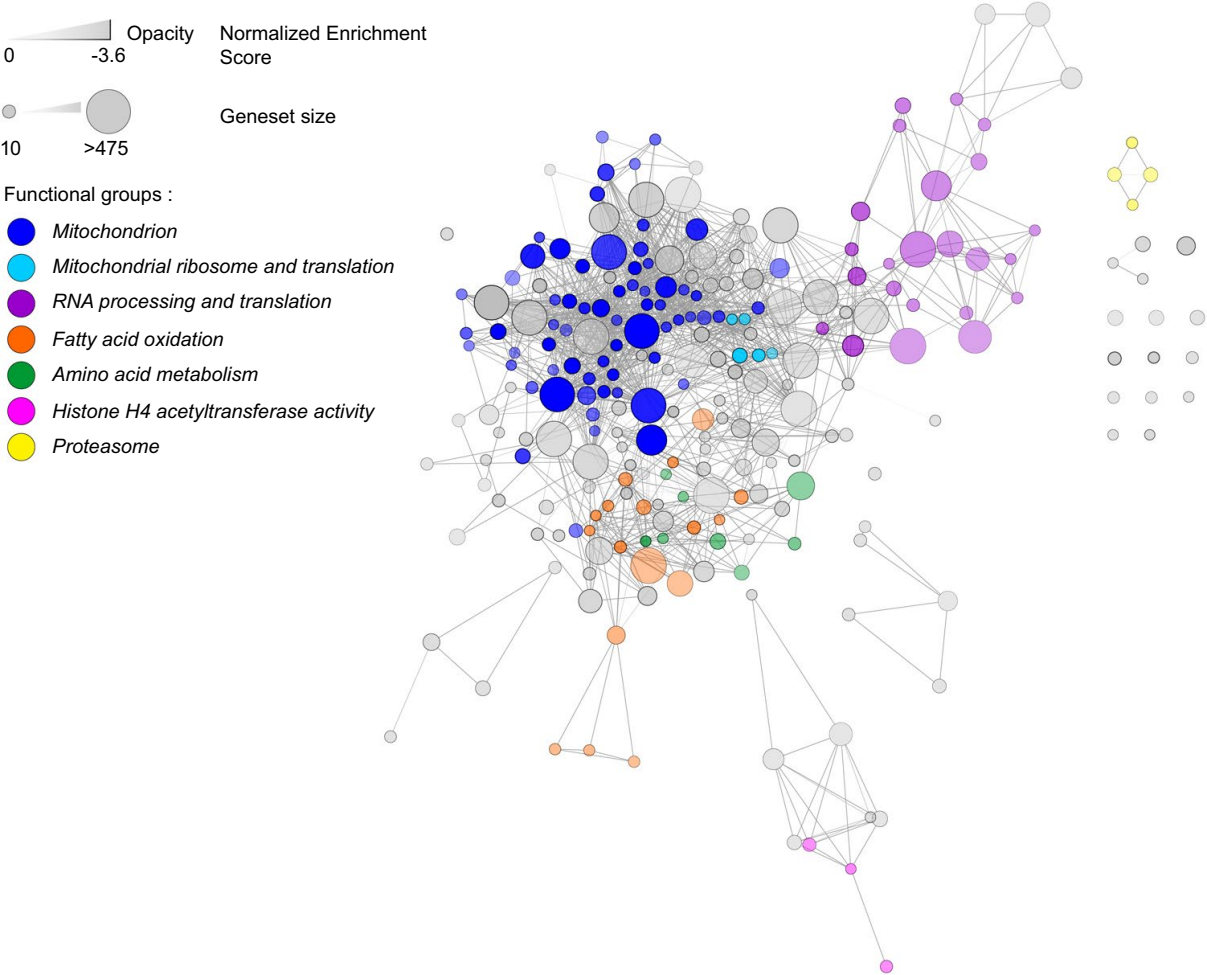
Network representing the genesets downregulated in the skeletal muscle of pre-frail subjects, organized by geneset similarity. Every node is a geneset that is significantly downregulated in pre-frail subjects. The size of the node is dependent on the size of the geneset. The nodes are connected depending on geneset similarity, i.e. on the number of genes that they have in common. The more opaque nodes correspond to the highest normalized enrichment score. Several functions were found more represented than others and are highlighted in color (Mitochondrion; Mitochondrial ribosome and translation; RNA processing and translation; Fatty acid oxidation; Amino acid metabolism; Histone H4 acetyltransferase activity; Proteasome). This figure shows that the Mitochondrion functional group is the most represented and the most downregulated in the prefrail muscle.

**Figure 4:**
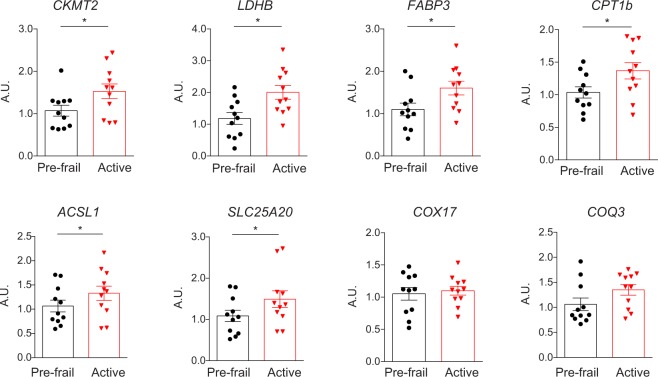
Gene expression in muscle biopsies measured by qPCR. Expression of genes, as measured by qPCR, that were in the top 100 of the most differentially expressed genes between pre-frail and active elderly, and in the genesets significantly downregulated in pre-frail subjects. Solute carrier family 25 member 20, *SLC25A20*; acyl-CoA synthetase long chain family member 1, *ACSL1*; carnitine palmitoyltransferase 1B, *CPT1B*; fatty acid binding protein 3, *FABP3*; coenzyme Q3 methyltransferase, *COQ3*; lactate dehydrogenase B, *LDHB*; creatine kinase, mitochondrial 2, *CKMT2*; and cytochrome c oxidase copper chaperone, *COX17*. Bargraphs represent mean ± SEM. ^∗^*P* < 0.05 after a two-tailed Student *t* test.

Gene expression in skeletal muscle was compared using Affymetrix HTA2.0 microarray. Data were analyzed running a Gene Set Enrichment type of Analysis (GSEA), which tells which biological processes are up or down-regulated at the scale of entire gene-sets, rather than gene by gene^15^. Results were filtered by selecting genesets up- or down-regulated with a false discovery rate (FDR) equal or less than 0.1. In total, there were 307 genesets significantly differentially expressed between the two groups, with 298 downregulated genesets and 9 upregulated genesets in pre-frail compared to active subjects (Table S1). Downregulated genesets in pre-frail subjects were related to RNA processing and translation, histone H4 acetyltransferase activity, proteasome, fatty acid oxidation, amino acid metabolism, and a majority to mitochondrion and the respiratory chain (Fig. 3). In fact, the 10 top downregulated genesets in pre-frail subjects were all related to mitochondrion (Table 4). Finally, the genes that were present in these genesets were compared to the top 100 of the most differentially expressed genes between the pre-fail and the active subjects. A short list of eight genes was selected and tested by qPCR, including solute carrier family 25 member 20 (*SLC25A20*), acyl-CoA synthetase long chain family member 1 (*ACSL1*), carnitine palmitoyltransferase 1B (*CPT1B*), fatty acid binding protein 3 (*FABP3*), coenzyme Q3, methyltransferase (*COQ3*), lactate dehydrogenase B (*LDHB*), creatine kinase, mitochondrial 2 (*CKMT2*), and cytochrome c oxidase copper chaperone (*COX17*). Amongst these, genes encoding proteins related to fatty acid oxidation (*CPT1B* and *FABP3*), Coenzyme Q10 synthesis (*COQ3*), anaerobic glycolysis (*LDHB*), and creatine phosphorylation (*CKMT2*) had a significantly lower level of expression in the pre-frail samples (Fig. 4).

**Table 4:** Top 10 down-regulated pathways in the skeletal muscle of elderly pre-frail.

GENESET NAME	ES	NES	FDR
GO_CELLULAR_RESPIRATION	−0.7709094	−3.813652	<1 × 10^−6^
GO_MITOCHONDRIAL_MEMBRANE_PART	−0.7268397	−3.695907	<1 × 10^−6^
GO_MITOCHONDRIAL_PROTEIN_COMPLEX	−0.7401110	−3.666778	<1 × 10^−6^
GO_OXIDATIVE_PHOSPHORYLATION	−0.7954150	−3.560389	<1 × 10^−6^
GO_INNER_MITOCHONDRIAL_MEMBRANE_PROTEIN_COMPLEX	−0.7571530	−3.549975	<1 × 10^−6^
GO_ORGANELLE_INNER_MEMBRANE	−0.6057178	−3.547871	<1 × 10^−6^
GO_RESPIRATORY_CHAIN	−0.7788278	−3.544574	<1 × 10^−6^
GO_ELECTRON_TRANSPORT_CHAIN	−0.7635362	−3.467508	<1 × 10^−6^
GO_AEROBIC_RESPIRATION	−0.8388420	−3.463427	<1 × 10^−6^
GO_MITOCHONDRIAL_PART	−0.5713046	−3.439396	<1 × 10^−6^

ES: enrichment score; NES: normalized enrichment score; FDR: false discovery rate.

## Discussion

Pre-frailty is the stepping stone to geriatric disorders that restrict mobility such as the frailty syndrome and sarcopenia, and intervening at this stage by targeting key biological pathways may have important implications in either reversing pre-frailty or halting the subsequent development of frailty. The goal of our clinical study was to characterize a pre-frail elderly population with respect to their mitochondrial function status using a comprehensive set of *ex vivo* and *in vivo* methods. The results reported in this manuscript demonstrate a striking association of the development of pre-frailty with a decline in skeletal muscle mitochondrial function.

When comparing with the existing literature, a comprehensive evaluation of mitochondrial function status in a well-characterized and clinically relevant population such as the pre-frail elderly is generally lacking. Our study is differentiated from these published studies on three aspects: a) a careful clinical selection of a pre-frail Dutch population using the very well defined Fried frailty criteria in the clinic; all pre-frail subjects met at least 2 criteria of low muscle strength and low physical activity; b) a comprehensive evaluation of mitochondrial function status using both *in vivo* (^31^P-MRS) and *ex vivo* on muscle biopsy samples (protein and genomic expression) methods, and c) comparing the pre-frail to an age-matched, physically very active group of elderly subjects with robust muscle strength. This last point was key in order to stratify the two studied elderly populations based on muscle strength and physical activity levels with the chosen number of participants. One of the limitations of the current study is that having a third group of frail elderly (3 or more Fried criteria) would perhaps have made the current study even more robust. We elected to focus on the pre-frail subjects only, also as we felt it was most relevant to understand the biology linked to the onset of the decline in mobility during aging.

Only a few studies have employed similar techniques to carefully assess muscle mitochondrial function, though inconsistent inclusion criteria have been employed to differentiate “low” *vs* “high”-functioning elderly, and the early pre-frail stage has not been a focus of these investigations. Elderly population differ depending on these inclusion criteria, focused on either aerobic endurance capacity (VO_2_ peak), physical performance scores (SPPB), self-reported physical activity levels, muscle strength or mass. Overall, “low”-functioning elderly present a lower mitochondrial function, compared to “high”-functioning subjects, when the two populations are separated by VO_2_ peak^14,16^, self-reported habits of exercise^11,17^ and muscle strength^18^. On the contrary, there is no difference in muscle mitochondrial function when populations have similar levels of physical activity, but are distinguished based on muscle mass^13^ or SPPB score^19^. This reinforces the hypothesis that lower mitochondrial function is tightly coupled to low physical capacity and low muscle strength in elderly.

Decrease in mitochondrial capacity in the muscle can be explained by a diminution in the number of mitochondria, their volume and/or their energetic yield. In the present study, pre-frail subjects had significantly less abundant respiratory complexes I, IV and V, lower activity of complexes I, II and IV, and trend for a lower mtDNA/nuDNA. This was accompanied by a striking lower transcription level of mitochondrial gene sets in muscle. Likewise, lower abundance of complex I, IV and V, a lower mitochondrial volume density as determined by EM, and a lower mitochondrial function was also reported in muscle of sedentary *vs* active elderly^11,17^.

With regard to the microarray data, to the best of our knowledge, we were not able to find a similar study where muscle transcriptome was compared in pre-frail *vs* active elderly. Only two studies reported a significant increase in the transcript levels of mitochondrial genes in muscle of elderly either after a 3-month of exercise training at 80% of maximal VO_2_^20^ or a 6-month resistance-training program^21^. A common signature that is redundant between these studies and ours is the upregulation of fatty acid oxidation related genes, such as *FABP3* and *CPT1B*, in active elderly or after an exercise intervention compared to sedentary elderly^21^. Likewise, induction of these fatty acid oxidation related genes was also described in studies where transcripts in muscle of young subjects were compared before and after exercise, or between sedentary and athletic subjects^22–25^.

Today, there are limited interventions for the management of age related muscle decline and associated geriatric conditions such as pre-frailty, frailty and sarcopenia. Pharmacologically, most interventions have focused on improvements in muscle mass either via selective androgen receptor modulation^26,27^, testosterone replacement therapy in men^28^ or with blocking antibodies targeting myostatin inhibition to improve muscle function^29^. The limitations in these approaches lie in the side effects of the different molecules or their route of administration, and the fact that they are rather used at late stages of muscle function decline such as frailty or sarcopenia. When prevention is still possible, optimal nutrition, especially a high protein diet along with resistance and aerobic exercise protocols is recommended to slow down the progression of these conditions^30^. However, adherence to exercise regimens and the quality of protein intake often dictates the outcome of these interventions in elderly. A complementary approach could integrate the current innovations in nutrition to help optimize mitochondrial function. In fact, several ingredients derived from food are being investigated to alleviate the burden of conditions associated with mitochondrial dysfunction, including L-carnitine^31–33^, epicatechin^34^ (contained in green tea) and nicotinamide riboside^35–38^ (present in beer and dairy products). Another interesting candidate is the newly discovered inducer of mitophagy, urolithin A, a gut metabolite derived from foods containing ellagitannins, such as pomegranate, red berries and nuts^39^. In fact, urolithin A was demonstrated to improve muscle and mitochondrial function in both young rats and aged mice^40^. These results are consistent with the hypothesis that a decrease in autophagy and mitophagy plays a key role in the decline of mitochondrial and muscle function observed during aging^41^.

In conclusion, results reported in this manuscript highlight the need to intervene early in the decline of muscle function and call for an integration of exercise, dietary interventional strategies and pharmaceutical approaches to boost mitochondrial function to manage muscle health in the elderly.

## Materials and Methods

### Trial design

The study was conducted as a single center, observational and case-control, clinical study. It was approved by an independent human ethics committee Stichting Bebo (Assen, the Netherlands) and conducted in accordance with the principles of the Helsinki Declaration. Informed consent was obtained from all the subjects. Adverse events and concomitant medications were continuously registered throughout the entire study period. The study was registered in a clinical trial registry as NCT02472340 (clinicaltrials.gov).

### In- and exclusion criteria

General inclusion criteria included an age of 61 years or higher; body mass index between 15–35 m^2^/kg. Additional inclusion criteria for pre-frail subjects were derived from the Fried frailty criteria^3^. Subjects were considered pre-frail when fulfilling one to two of the following three criteria: either a walking speed <0.8 m/s in the 4-m walking test, or a low muscle mass (SMI for males <10.75 kg/m^2^, for females <6.75 kg/m^2^) or a low muscle strength (handgrip strength of <30 kg for males and <20 kg for females); and sedentary behavior, which was defined as having an activity category of 1 (<600 MET (metabolic equivalent unit) per week). Additional inclusion criteria for active subjects included an activity level of category 2 or 3 (≥600 MET per week); normal gait speed (a walking speed ≥ 0.8 m/s in the 4-m walking test); normal skeletal muscle mass (SMI for males ≥10.75 kg/m^2^, for females ≥6.75 kg/m^2^); normal muscle strength (handgrip strength of ≥30 kg for males and ≥20 kg for females).

General exclusion criteria included any contraindication to have a MRI scan; diabetes mellitus, any underlying chronic disease and/or lower extremity peripheral vascular disease; a history (within 3 months of screening) of alcohol consumption exceeding 2 standard drinks per day on average; inability to refrain from smoking more than half a pack of cigarettes (or similar for other tobacco products) per day during the course of the study; alcohol consumption within 48 hours of the study visits; unwillingness or inability to have a muscle biopsy performed; a history of allergy to lidocaine; and participation in a clinical trial within 90 days of screening or more than 4 times in the previous year. Participants were also advised to refrain from consuming muscle health promoting supplements two weeks prior to study enrollment. Additional exclusion criteria for pre-frail subjects included ruling out cachexia i.e. unintentional weight loss ≤5% of usual body weight during the last 6 months; anorexia; and anorexia-related symptoms.

### Study schedule

Subjects were screened for eligibility up to 45 days before study enrollment. The tests used to identify eligible subjects included International Physical Activity Questionnaire (IPAQ), body composition by bioelectrical impedance analysis, Short-Physical Performance Battery Test (SPPB) and handgrip strength by Jamar dynamometry (Table 2). From the SPPB, only the walking speed was used as an inclusion criteria.

Upon study enrollment, subjects were tested at day 1 and day 14 for 1) SPPB, 2) handgrip strength by Jamar dynamometry, 3) quadriceps strength, 4) postural stability and 5) *in vivo* evaluation of mitochondrial function using phosphorus magnetic resonance spectroscopy (^31^P-MRS) (Table 2). Muscle tissue collection and blood sampling for *ex vivo* biological markers of mitochondrial function were only performed at the day 1 visit to limit the burden on the subjects. The muscle biopsy procedure was overall well tolerated. These biopsies were used to assess mitochondrial function *ex vivo*, by measuring mitochondrial respiratory complexes activity and abundance, and mtDNA/nuDNA ratio. Subjects returned to the clinical research unit for the removal of the suture and inspection of the wound from the muscle biopsy procedure on day 7. Subjects were contacted by telephone for follow-up 7 to 10 days after the day 14 visit.

### Physical performance measurements

#### International Physical Activity Questionnaire (IPAQ)

The Dutch version of the IPAQ was used to estimate an individual’s level of physical activity in the domains of household and yard work activities, occupational activity, self-powered transport, and leisure-time physical activity as well as sedentary activity. The long version was used to gain a more detailed insight in the level of activity. An additional question asked about the pace of walking and cycling. The questionnaire and its translation into the Dutch language has been validated by direct comparison to activity measurements using an accelerometer^42^. The questionnaire was only taken during screening.

#### Bioelectrical impedance analysis

Bioelectrical impedance analysis has been found to correlate well to Dual Energy X-ray Absorptiometry (DEXA) and to Magnetic Resonance Imaging (MRI) in estimating muscle mass and body composition measurements and is therefore a validated part of the diagnostic work-up in elderly populations^43–45^. Using the InBody720 body composition analyzer (Biospace Co., Ltd., Korea), the Skeletal Muscle Mass index (SMI) was derived as part of the screening. The SMI (in kg/m^2^) was calculated by dividing the lean muscle mass (in kg) by the square body height (in m^2^). The SMI was only performed during screening.

#### Short Physical Performance Battery (SPPB)

The short physical performance battery (SPPB) has been described as a group of measures that combines the results of the gait speed, chair stand and balance tests^46^. It has been used as a predictive tool for possible disability and can aid in the monitoring of function in older people^47^. The scores ranged from 0 (worst performance) to 12 (best performance). We tested the ability to maintain standing balance during 10 seconds in three different positions: side-by-side, semi-tandem and tandem stance. A score of one was attributed to each of the position if the balance was maintained successfully. We also measured walking speed during the 4-meter walking test and the timed sit-to-stand transfer test. The test instructions have been described by Guralnik *et al*.^46^.

#### Grip Strength

Grip strength was measured using the Jamar Plus dynamometer device (Patterson Medical, Nottinghamshire, United Kingdom). Each subject was positioned in a straight-backed chair with both feet placed flat on the floor. Grip strength (in kg) was determined in the dominant hand. Subjects were instructed to keep an upright posture, with the elbow flexed at 90° and the forearm and wrist in neutral position. The subject was verbally motivated to provide maximum grip force. Measurements were performed three times per occasion by the same investigator and the highest force was used for further analysis.

#### Quadriceps Strength

Maximal voluntary strength testing of the right quadriceps muscle was assessed using a handheld dynamometer (CITEC, type CT 3001, C.I.T. Technics, Haren, The Netherlands). Such devices have previously been utilized and validated for lower limb muscle strength testing^48^ and correlate well with other methods such as isokinetic strength testing to assess lower limb muscle strength^49^. The subjects were laying on an examining table in prone position with the right knee flexed to a 90° angle with the dynamometer placed against the instep. Maximal voluntary strength in the quadriceps was exerted by extending the knee joint with the investigator keeping the position of the dynamometer fixed. Subjects were verbally motivated to apply maximal voluntary eccentric force^50^. Measurements were performed three times per occasion by the same investigator and the highest force was used for further analysis.

#### Postural stability

The sway of a subject was assessed in a single, horizontal plane. A string was attached to the subject’s belt to measure postural stability with closed eyes to exclude the influence of vision on postural control^51^. The total amount of sway (in mm) was measured over a time period of 2 minutes.

### *In vivo* mitochondrial function measurements

#### 31 phosphorus – Magnetic Resonance Spectroscopy (^31^P-MRS)

^31^P-MRS scanning to determine mitochondrial function *in vivo* has been widely employed in characterizing the mitochondrial function status in multiple disease populations^13,52–55^. Dynamic ^31^P-MRS was performed on a 7 tesla human MRI scanner (Phillips, Best, The Netherlands) on the right posterior calf using a custom-built 8 × 6 cm ^31^P-surface coil during exercise on a specially-designed MRI-compatible pedal. Subjects were tested in the morning in the fed state. The pedal was designed to allow the study subjects to perform isometric plantar flexion exercise by pressing against a foot pedal while lying in the supine position. The right foot was strapped firmly to the exercise device using non-elastic Velcro straps proximal to the base of the fifth digit with the right knee supported. The subject’s lower extremity was secured to the MRI table with straps across the mid-thigh and mid–lower leg in order to isolate usage of the posterior calf muscles. Subjects were instructed to sub-maximally flex the right foot in order to contract the calf muscle and thus to decrease the phosphocreatine levels, which could be monitored real time by the investigator. Muscle force was built up over the exercising period up to maximal voluntary contraction. The subjects performed plantar flexions for a maximum of 3 minutes with rest intervals between (2.5 seconds exercise, 1.5 seconds rest). The scanning protocol consisted of localizer sequences and the acquisition of a field map for shimming purposes using a custom-built outer partial volume coil tuned to the proton frequency. Thereafter, ^31^P MRS data were acquired before, during and after exercise with a time resolution of 1 second. Peak integrals of the inorganic phosphate (Pi) and PCr signals were obtained using the jMRUI software package (version 5.0, jMRUI Consortium). The frequency difference between PCr and Pi was used to calculate tissue pH. Scans with an end-of-exercise pH of <6.8 were excluded from the analysis, as determination of the PCr recovery rate in this situation is unreliable^56^. In such a case, subjects were rescanned once with a minimal time of 15 minutes in between scans. Mitochondrial function was determined by plotting the PCr peak integrated area against the time during exercise recovery^57^. Recovery curves were fitted to a mono-exponential function to determine the PCr recovery rate (τPCr) using a custom made MatLab script (version 2012b). τPCr recovery rates were compared between the occasions. Outliers were manually removed using the MatLab script.

### *Ex vivo* biomarkers for mitochondrial function

#### Muscle biopsy procedure

Muscle biopsy were performed around noon, after a 5 hours fasting period following breakfast. Tissue was collected from the *vastus lateralis* muscle of the right leg using a 4.5 mm Bergström muscle biopsy needle (Maastricht Instruments, Maastricht, The Netherlands). The subject was placed in a semi-supine position with the knees supported and slightly flexed. The lateral side of the leg was palpated to determine the location of biopsy, 10 cm proximal of the upper pole of the patella on a line between the patella and the anterior superior iliac spine. After disinfecting the skin, the skin and muscle fascia were locally anaesthetised with a 5 mL lidocaine 5% solution. More lidocaine (up to 10 mL) was administered when the anaesthetic effect was not sufficient. During all procedures it was ensured that the lidocaine did not infiltrate the muscle. A sterile cloth with a hole was placed on the leg, keeping the biopsy site exposed. A small incision of 5 mm was made in the skin and the muscle fascia was incised minimally, just wide enough for the biopsy needle to pass through. The biopsy needle was introduced via the skin and fascia into the muscle. A vacuum was applied on the needle using a sterile 20 ml syringe to increase the muscle yield^58^. Immediately after collection the samples were weighed to determine if more tissue was required. After collecting the required amount of muscle tissue, the wound was closed with a single non-absorbable skin suture and pressure was applied by an elastic bandage. Subjects were instructed not to perform strenuous physical activity with the right leg for two days. The suture was removed after 7 days at the clinical research unit. Tissue collected for DNA quantification and protein analysis was snap frozen in liquid nitrogen within 30 minutes of collection and stored at −80 °C. All the analysis performed on muscle biopsies were done in a blinded fashion by independent laboratory personnel and only the initials of the subject and date of collection of muscle biopsy samples were listed on the samples.

#### Preparation of protein lysates from muscle biopsies

Frozen tissue samples were embedded in Cryoembedding Medium (Medite, Germany) and cut into 20 μm sections using a CryoStar NX70 cryostat (Fisher Scientific, Schwerte). For protein analysis by ELISA, tissue slices were incubated on ice for 30–60 min in 1xLM lysis buffer from NADH dehydrogenase (Complex I) Human SimpleStep^TM^ ELISA kit (Abcam, ab178011). Buffers were added in complete mini Protease Inhibitor Cocktail (Roche, Germany), HALT Protease inhibitor (Fisher Scientific, Germany) and Phosphatase inhibitor cocktails 2 and 3 (Sigma, Germany). Lysates were vortexed before centrifugation for 10 min at 13,000 × g to precipitate tissue debris. Supernatants were aliquoted and stored at −80 °C until analysis. Protein concentration was determined using the bicinchoninic acid (BCA) protein assay (Sigma, Germany).

#### Quantification of mitochondrial respiratory complexes abundance and activity by ELISA in muscle biopsies

Protein lysates were analyzed for complex I abundance using the NADH dehydrogenase Human SimpleStep^TM^ ELISA kit (Abcam, ab178011), for complex IV abundance using the cytochrome c oxidase Human SimpleStep^TM^ ELISA kit (Abcam, ab179880) and for complex V using the ATP synthase Human Profiling ELISA kit (Abcam, ab124539). Mitochondrial respiratory complex activity was determined using the Complex I enzyme activity microplate assay kit (Abcam, ab109721), the Complex II enzyme activity microplate assay kit (Abcam, ab109908) and the Complex IV human enzyme activity microplate assay kit (Abcam, ab109909). Protein abundance or activity were determined using the linear range of a standard curve made with HeLa cells lysates (complexes I and IV abundance), with HepG2 cells lysate (complex V abundance) or bovine heart mitochondrial lysate (BHM) (Abcam, ab110338) (complexes I, II and IV activity). All assays were performed following the manufacturer’s instructions. In addition, all assays were validated to ensure that the signal was in the linear range of the detection system.

#### DNA extraction

Muscle samples were incubated overnight in 360 µl of buffer ATL and 40 µl Proteinase (Qiagen, USA) at 55 °C in a thermomixer set at 300 rpm. Cell debris were removed by centrifugation and 200 µl of clear lysates were placed in the QIAsymphony SP workstation (Qiagen, USA). DNA was extracted with the QIAsymphony DNA Mini kit (Qiagen cat# 937236) following manufacturer’s procedures.

#### Quantitative PCR analysis

Quantitative PCR was performed on the Fluidigm Biomark system following the Fluidigm Specific Target Amplification Quick Reference (Fluidigm, USA). Samples were loaded as technical triplicates. The real-time PCR data were analyzed using the Linear Derivative baseline correction and User (detector) Ct threshold method on the latest version of the Fluidigm BioMark software (ver. 4.1.3). Quantification of mitochondrial DNA (mtDNA) was performed using two customized Taqman assays targeted against a nuclear DNA sequence (*RNA18SN1*) and a conserved region of mtDNA (*MTND1*)^59^. Relative mtDNA copy number was determined comparing *MTND1* to *RNA18SN1* signal. All quantifications were determined using the 2^−∆∆Ct^ method and the mean Ct of the technical triplicates.

#### RNA extraction and cDNA synthesis

Muscle samples were homogenized in 800 µl of buffer RLT plus (Qiagen, USA) plus 2 steel balls using a Tissue Lyser (Qiagen, USA). Cell debris were removed by centrifugation and clear lysates were placed in the QIAsymphony SP workstation (Qiagen, USA). RNA was extracted with the QIAsymphony RNA kit (Qiagen cat# 931636) following manufacturer’s procedures.

RNA was quantified and checked for purity on a Nanodrop-8000. RNA integrity was controlled using RNA 6000 Nano LabChip kit (Agilent technologies cat# 5065-4476) on an Agilent Bioanalyzer (Agilent technologies).

#### Analysis of gene expression by microarray

2 ng of total RNA was run on a GeneChip® Human Transcriptome Array 2.0 (Affymetrix) after cDNA synthesis following manufacturer’s instructions. The array was read on a GeneChip 3000 Scanner (Affymetrix). The data were normalized with the SST-RMA method (SST = Signal Space Transformation; RMA = Robust Multi-array Average).

#### GSEA method

All the analysis were done using the R statistical programming language^60^ program and Bioconductor^61^ R libraries. The genes were ordered in a ranked list according to the magnitude and direction of their differential expression between the pre-frail and active groups using the limma package. Genesets used for the GSEA analysis were taken from the Human Gene Ontology (GO) categories (http://www.geneontology.org/). Genesets were considered significantly up- or down-regulated with a false discovery rate (FDR) ≤0.1. Network representation of the significantly downregulated genesets in pre-frail subjects was based on the similarity coefficient (i.e. number of genes in common between two genesets) and was done using Cytoscape 3.4.0.

### Statistical analysis

This is an exploratory study and as such the subject number was based on previously published studies conducted in sedentary elderly^11^. To establish whether significant effects could be detected on the repeatedly measured pharmacodynamic parameters, each parameter was analyzed with a mixed model analysis of covariance (ANCOVA) with treatment, time, sex and the interactions as fixed factors and subject as random factor and the (average) baseline measurement as covariate. Pre-frail and active subjects were matched in pairs based on age, gender and physical activity status. The Kenward-Roger approximation was used to estimate denominator degrees of freedom and model parameters are estimated using the restricted maximum likelihood method. For the physical performance tests, statistical significance was computed on the screening values for the skeletal muscle index and for the level of physical activity assessed by IPAQ. For all the other parameters that were measured at day 1 and day 14, the statistical significance was determined between the two groups on the averaged values between day1 and day 14. The general group effect and specific contrasts were reported with the estimated difference and the 95% confidence interval, the Least Squares Means (LSM) estimates and the p-value. All calculations were performed using SAS for windows V9.4 (SAS Institute Inc., Cary, North Carolina, United States).

### Data availability

The genomic dataset generated during the current study are not publicly available due to intellectual property purposes but are available from the corresponding author on reasonable request.

## Electronic supplementary material

Table S1
