# Publisher Correction: Mitochondrial function is impaired in the skeletal muscle of pre-frail elderly

**DOI:** 10.1038/s41598-019-54822-7

**Published:** 2019-11-25

**Authors:** Pénélope A. Andreux, Marcus P. J. van Diemen, Maxime R. Heezen, Johan Auwerx, Chris Rinsch, Geert Jan Groeneveld, Anurag Singh

**Affiliations:** 1 Amazentis SA, EPFL Innovation Park, Batiment C, CH-1015 Lausanne, Switzerland; 2 Center for Human Drug Research, Zernikedreef 8, 2333 CL Leiden, The Netherlands; 3 Laboratory for Integrative and Systems Physiology, Ecole Polytechnique Fédérale de Lausanne, CH-1015 Lausanne, Switzerland

Correction to: *Scientific Reports* 10.1038/s41598-018-26944-x, published online 04 June 2018

In the original version of this Article, the author Geert Jan Groeneveld was incorrectly indexed. This error has now been corrected.

